# A case report of diagnosis and dynamic monitoring of *Listeria monocytogenes* meningitis with NGS

**DOI:** 10.1515/biol-2022-0738

**Published:** 2023-11-08

**Authors:** Jiamei Jiang, Meng Lv, Kaichao Yang, Gang Zhao, Yimu Fu

**Affiliations:** Department of Emergency Medicine, Shanghai Jiaotong University Affiliated Sixth People’ Hospital, No. 600 Yishan Road, Xuhui District, Shanghai 200233, China; Genoxor Medical Science and Technology Inc., Shanghai 201100, China

**Keywords:** metagenomic next-generation sequencing, targeted next-generation sequencing, *Listeria monocytogenes*, *Listeria monocytogenes* meningitis

## Abstract

*Listeria monocytogenes* (*LM*) infections of the central nervous system are deadly and have vague symptoms. Traditional cerebro spinal fluid culture has a low positive rate, and because antibiotic use is common following therapy, it is more challenging to assess the response from pathogen content. In this case, a 66-year-old man who had a fever, a headache, and vomit was admitted to the hospital. He had diabetes, decline in thyroid function, and a history of pituitary tumor removal surgery. His initial treatment with ribavirin, ceftriaxone antibiotic, and moxifloxacin did not go well. Using two etiological tests (culture and metagenomic next-generation sequencing [mNGS]), his cerebrospinal fluid tested positively for *LM*. Ampicillin-sulbactam and meropenem were used as treatments once *LM* meningitis was identified. After treatment, his cerebrospinal fluid was assessed once more. Culture: negative; targeted next-generation sequencing (tNGS): positive and shows changes in the copy number of the *LM*. After 44 days of treatment, the patient finally stopped taking antibiotics, and the prognosis was good. Our study showed that mNGS and tNGS, as novel approaches for pathogen detection, are capable of identifying pathogens quickly, sensitively, and accurately, especially when there are few infections present (such as after antibiotic treatment). The two methods can be a powerful assistance for helping clinicians to choose the best course of action.

## 1 Background

*Listeria monocytogenes* (*LM*) is a gram-positive, facultatively anaerobic, intracellular bacteria extensively present in the environment [1]. Since the 1980s, it has been responsible for foodborne illness outbreaks and is the cause of listeriosis. In Europe, the prevalence of *LM* is estimated to be around 0.48 cases per 100,000 individuals, and infections can manifest either sporadically or in an epidemic manner. According to the Centers for Disease Control and Prevention (CDC), the yearly occurrence of laboratory-confirmed listeriosis in the United States is approximately 0.24 cases per 100,000 individuals. Various species of wild and domestic animals, including mammals and birds, have the potential to acquire *LM* infection, thereby serving as potential zoonotic reservoirs for this pathogen [2]. Animals usually undergo the infection asymptomatically, and its result may be contamination of food of animal origin, mainly meat and milk. Consuming contaminated food is the most common way to make people sick. Large amounts of infected food taken by a healthy host are unlikely to induce anything more than self-limiting febrile gastroenteritis [3,4]. However, it is a serious bacterial infection for those with immunosuppression, children, the elderly, pregnant women, and others [5]. In these people, there is an increase in symptoms such as fever, chills, muscle pain, headache, nausea, and vomiting, and it is associated with invasive infection and high mortality. *LM* mainly causes central nervous system (CNS) infections (meningitis or meningoencephalitis) and systemic infections.

Metagenomic next-generation sequencingr (mNGS) has demonstrated considerable promise for identifying infections of the CNS [6,7]. Numerous case studies show how mNGS can be used as a novel method to simultaneously and reliably detect *Listeria monocytogenes* meningitis (LMM) [8–11]. Targeted next-generation sequencing (tNGS), another sequencing method used in clinical microbial identification in addition to mNGS, is mostly immune to interference from the human genome and colonizing bacteria [12]. It is effective for identifying infectious diseases with low copy numbers that are well-known and widespread [13]. In this case, tNGS was applied for monitoring patient outcomes following therapy, while mNGS was utilized for recognizing patients with LMM. Together, the two methods provided enough etiological evidence to justify the development of treatment regimens.

## 2 Case presentation

### 2.1 Investigations

A 66-year-old man with “3 days of fever accompanied by severe headache for 1 day, vomiting, and lethargy for 10 h” was admitted to our hospital’s internal medicine observation room in the afternoon of February 10, 2023. The patient had a history of diabetes for more than 10 years (Insulin Lispro injection, 24 u in the morning and 22 u in the evening) and hypothyroidism for 10 years (sodiumlevothyroxine, 1#qd). The patient had a pituitary tumor removed in 2013, and cortisone acetate (25 mg qd) was administered postoperatively for a very long time. The patient experienced various symptoms without obvious causes 2 days before admission (February 7), including fever (up to 38.8°C), cough, less sputum, and other symptoms. After taking paracetamol via self-administration, the body’s temperature dropped. The patient started experiencing severe headache and a lingering fever (body temperature of 38.2°C) on February 9. The patient’s headache slightly lessened following moxifloxacin (100 mg qd) and ibuprofen (0.6 g qd) treatment in our hospital’s fever clinic (Table 1).

**Table 1:** History of microbiological testing and treatment

Date	Hospital stay (days)	Microbiological testing	Result	Treatment
9 Feb	0	—	—	Moxifloxacin 100 mg qd
10 Feb	1	—	—	Ceftriaxone 2 g qd
11 Feb	2	Antibody testing	—	Ribavirin 0.5 g q8h + Ceftriaxone 2 g qd
12 Feb	3	First CSF test: mNGS + culture	HSV-1 IgG(+), IgM(+)	Not adjusted
14 Feb	5	—	First CSF mNGS: (+) *L. monocytogene* (149 reads)	Ampicillin sodium and sulbactam sodium 3 g q6h + meropenem 1 g q8h
15 Feb	6	—	First CSF culture: (+) *L. monocytogene*	Not adjusted
19 Feb	10	Second CSF test: tNGS + culture	—	Not adjusted
21 Feb	12	—	Second CSF tNGS: (+) *L. monocytogene* (3 copies/mL)	Not adjusted
22 Feb	13	—	Second CSF culture (−)	Not adjusted
28 Feb	19	Third CSF test: tNGS		Not adjusted
1 Mar	20	—	Third CSF tNGS: (−)	Not adjusted
6 Mar	23	—	—	Not adjusted

Notes: HSV-1: herpes simplex virus type 1; CSF: cerebrospinal fluid; mNGS: metagenomics next-generation sequencing; *L. monocytogene*: *Listeria monocytogene*; +: positive; −: negative.

**Figure 1:**
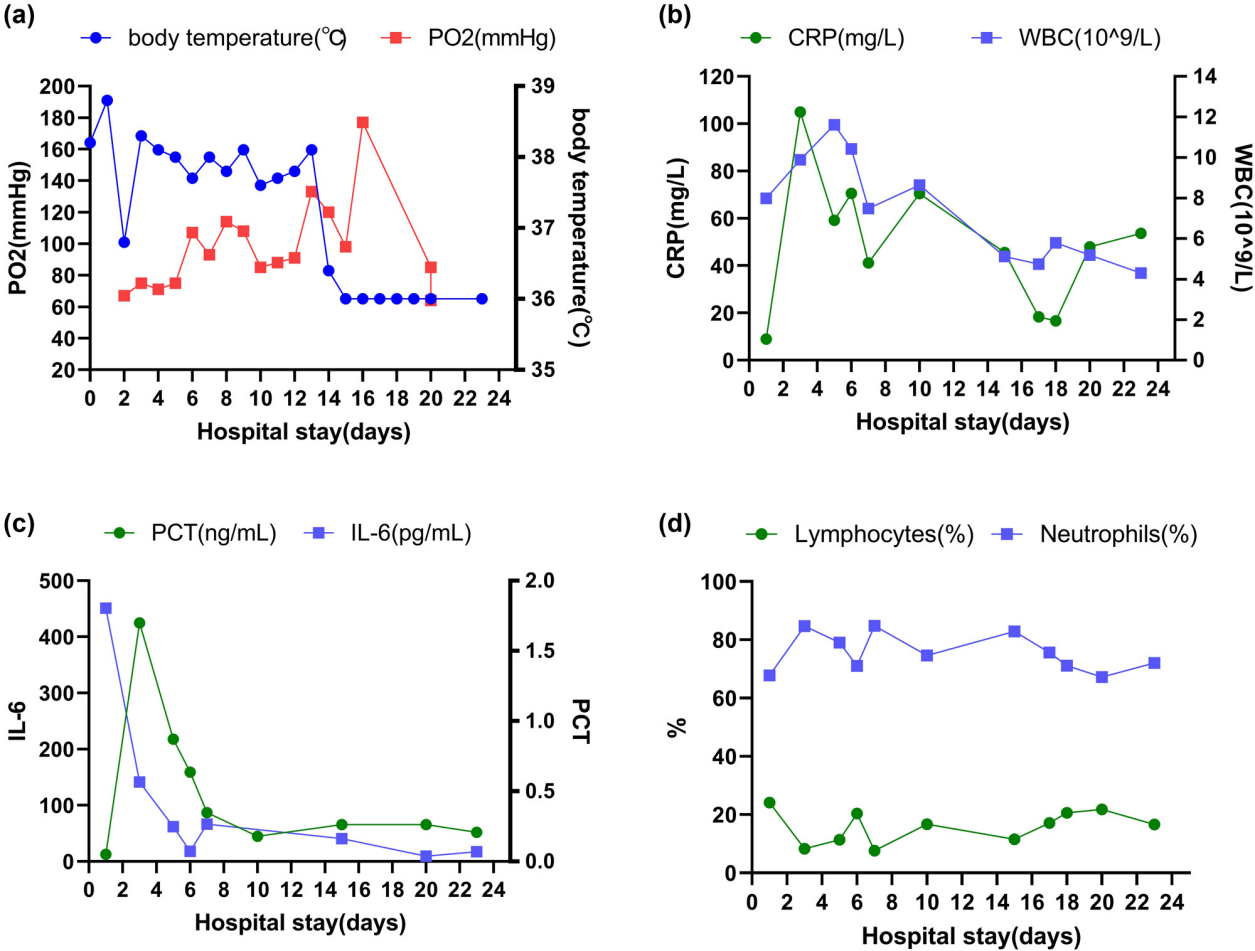
Dynamic changes in condition and blood tests after admission: (a) body temperature and partial pressure of oxygen (mmHg, PO_2_), (b) CRP and WBC (×10^9^), (c) PCT (ng/mL) and IL-6 (pg/mL), and (d) percentage of lymphocytes and neutrophils.

On the morning of February 10, the patient redeveloped headache, apathy, and ejected vomiting (stomach contents), but without limb twitches and hemisensory disturbances. Blood tests: C-reactive protein (CRP) 8.91 mg/L, white blood cell count (WBC) 7.98 × 10^9^/L, procalcitonin (PCT) 0.05 ng/mL, interleukin (IL)-6 451 (pg/mL), lymphocytes 24.1%, and neutrophils 67.8% (Figure 1). Chest computer tomography showed an infection site in the right lung. Ceftriaxone (2.0 g qd) was administered to the patient to begin anti-infective therapy (Table 1). However, the symptoms, which included chills, deteriorating consciousness, and shortness of breath, persisted, and the persistent temperature (38.6–39.6°C) did not go away.

### 2.2 Diagnosis and treatment

**Figure 2:**
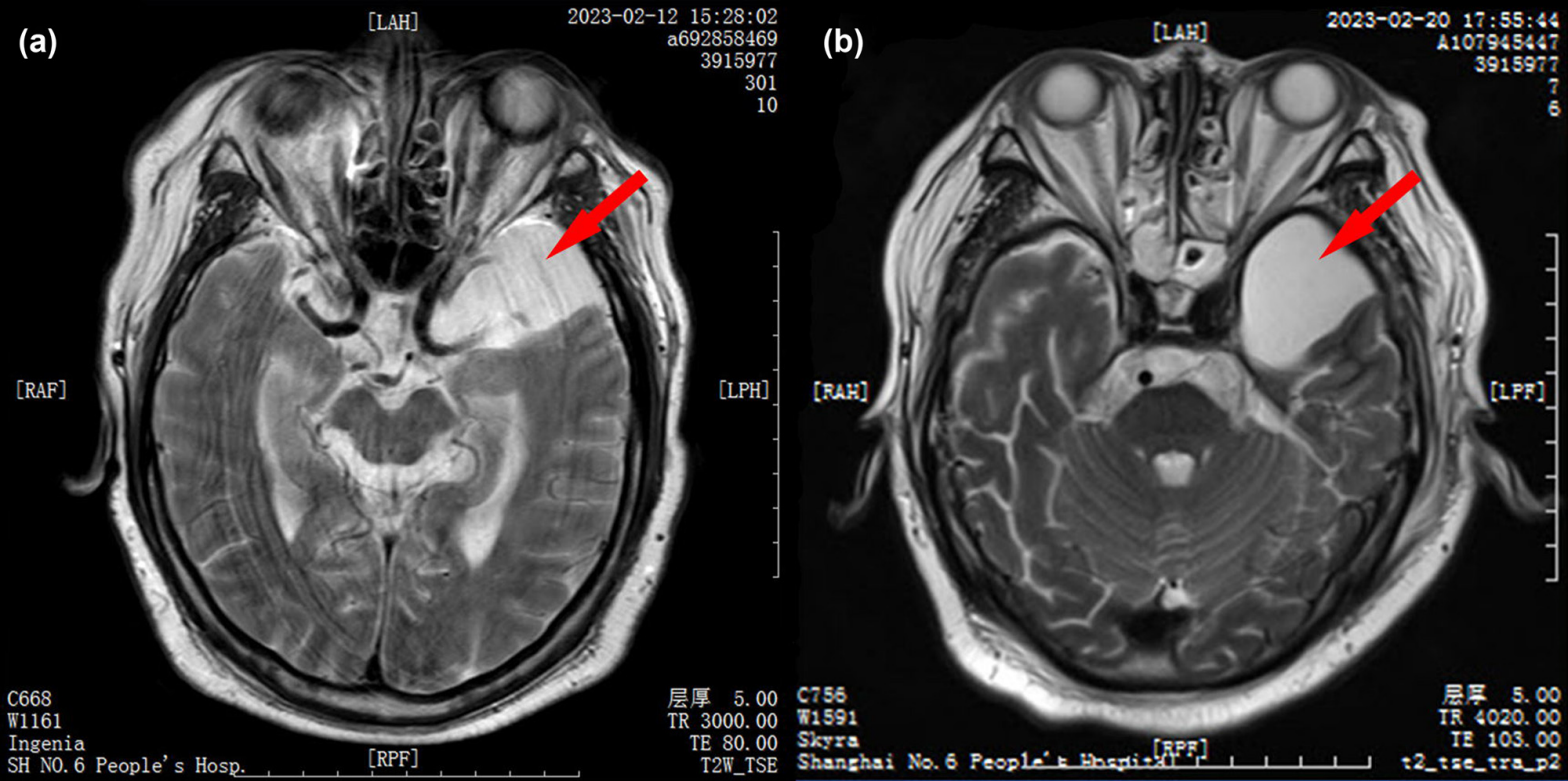
MRI demonstrated: (a) the third day after admission (12 February) and (b) Day 11 after admission (20 February). The results showed that *LM* infection did not cause parenchymal changes in the patient. The red arrow refers to the patient’s old cyst lesion.

The patient was transferred to our department (emergency intensive-care unit [EICU]) on the morning of February 12, and a lumbar puncture was carried out to obtain cerebrospinal fluid samples for the biochemical and microbiological testing (culture and mNGS, Table 1). The patient had a 38.6°C body temperature (Figure 1a), shortness of breath (40 breaths per min), restlessness, a glasgow coma scale score of 10, disorientation, and a double pupil of 2 mm at this time. As can be seen in Figure 1b–d, the blood test results were higher than the readings from February 10. The brain’s magnetic resonance imaging (MRI; Figure 2a) revealed a left temporal pole arachnoid cyst and mild bilateral frontoparietal lobe ischemia. Herpes simplex virus type I (HSV-1) IgG (+), IgM (+), peripheral blood antibody test, may be viral encephalitis. Thus ribavirin (0.5 g q8h) was added to the anti-infective therapy. However, the patient did not improve significantly. He experienced symptoms like breathlessness, unconsciousness, brief convulsions, and restlessness on February 13 and required invasive ventilator-assisted breathing therapy. On February 14, cerebro spinal fluid (CSF) biochemical and mNGS results were released. Cerebrospinal fluid biochemistry: protein 5.14 g/L, glucose 7 mmol/L, chloride 124 mmol/L; CSF PCR: *Mycobacterium tuberculosis* DNA (−). CSF mNGS results: *L. monocytogene* positive (149 reads). On February 14, after receiving his initial diagnosis of LMM, he stopped taking ceftriaxone right away and changed his anti-infection regimen to ampicillin-sulbactam (3 g q6h) + meropenem (1 g q8h) (Table 1). The CSF culture produced the same result as the mNGS (*L. monocytogene* positive) the following day (February 15). The mold was negative after 5 days of cultivation.

The patient continued to experience sporadic seizures and intermittent fever between February 14 and February 19 (Figure 1). CSF was collected for the second biochemical and microbiological test on the tenth day of admission (February 19) (tNGS and culture, Table 1). tNGS results: *L. monocytogene* (3 copies/mL) positive; culture: negative; CSF: leukocytes 620 × 10^6^/L, protein 4.14 g/L, glucose 9 mmol/L, chlorine 125 mmol/L. The patient’s condition had improved compared to when they first arrived in the EICU.

### 2.3 Outcome

The peak fever gradually decreased after February 19 and returned to normal on February 22 (Figure 1a). And the patient’s blood tests result gradually decreased (Figure 1b–d), consciousness gradually returned to normal, and the frequency of seizures decreased. On February 23, he was taken off the ventilator and put into an oxygen nasal cannula. However, the brain MRI on February 20 (Figure 2b) did not reveal any appreciable variations from February 12 (Figure 2a). We considered temporal lobe cysts on MRI as an ancient lesion in patient and did not believe it was connected to LMM. A third CSF NGS (second tNGS, February 28, Table 1) test was conducted before the patient was transferred and the result was negative. The final blood test (March 4, Figure 1b–d) results had obviously normalized before discharge.

On March 6, the patient was transferred to rehabilitation hospital to continue receiving anti-infective treatment (ampicillin sulbactam sodium, vancomycin, and ceftazidime; the dosage for each medicine is unknown). After 44 days of targeted antibiotic therapy (through March 30), the patient stopped taking antibiotics. In early May, the patient recovered and was discharged from hospital. Currently, the patient is resuming self-care.


**Informed consent:** Informed consent has been obtained from all individuals included in this study.
**Ethical approval:** The research related to human use has been complied with all the relevant national regulations, institutional policies and in accordance with the tenets of the Helsinki Declaration, and has been approved by the authors’ institutional review board or equivalent committee.

## 3 Methods

### 3.1 mNGS

The general detection process was as follows: (a) DNA extraction: the nucleic acid for the cfDNA libraries was extracted from 200 L of CSF. (b) RT-PCR and the 2100 Bioanalyzer equipment (Agilent Technologies, Inc.) were used to validate the library. The Nextseq CN500 was used for sequencing. (c) Bioinformatics analysis: first, removed low-quality tails and reads by Trimmomatic v0.36. Second, host reads (the reads mapping to the human reference genome GRCh37) were excluded using the short-read alignment tool Bowtie v2.2.6. Next, the remaining reads were aligned to the reference database, composing multiple public sequence resources of bacteria, viruses, and fungi.

### 3.2 tNGS

Total genome DNA extraction used the same procedures as mNGS. (a) Library preparation and enrichment: using the panel of 205 pathogens, the DNA was employed as a template for multiplex PCR amplification. Second, sequencing linkers and barcode sequences for sample identification were added to create pathogen sequencing libraries. Library concentrations were quantified using a Qubit 4.0 (Invitrogen). (b) Sequencing: high-throughput sequencing was performed using the Illumina Nextseq CN500 sequencing platform. (c) Bioinformatics analysis: first, the raw data obtained were filtering the low-quality data. Following identifying high-quality data by primer sequences, reads with correct paired-end overlap were compared with pathogen sequences from NCBI databases. The pathogen species and content in the samples can finally be identified.

## 4 Discussion

*LM* was first identified as a foodborne pathogen in the 1980s. It is a typical cold-tolerant bacteria that can grow at 4°C and is extremely resilient to salt and high temperatures [14]. CNS infections of *LM* consist of meningitis, encephalitis, and brain abscess [15,16]. The overall mortality rate was 30% [17]. An effective preventative strategy for vulnerable people is to steer clear of items that are often tainted with *LM*, such as soft-ripened cheeses, pate, prepared chilled meats, unpasteurized milk, and ready-to-eat chicken that has not been thoroughly cooked. In recent years, foodborne problems caused by Listeria and other pathogens have been frequent. In addition to avoiding contaminated food, many studies have proposed improving food quality [18]. For example, using natural substances with antioxidant and bactericidal effects (chia seeds, lemongrass essential oil, etc.) can help extend the shelf life of fruits, meat, dairy products, and others [19].

When infected with *LM*, timely diagnosis and appropriate early antibiotic therapy can significantly improve the patient’s survival rate (greater than 70%) [20]. However, only 15% of meningitis due to *LM* presents with the classical triad of meningitis (fever, headache, and nuchal rigidity) [21]. Therefore, the diagnosis of CNS infection caused by *LM* relies primarily on pathogen examination [5]. Bacterial culture is the “gold standard” for recognizing *LM* infections. However, the result of CSF culture might be affected by the rate of bacterial growth (which takes time) and the use of antibiotics [22,23]. Antibiotic use reduces bacterial burden and gradually lowers the percentage of cultures that are positive [24].

NGS has developed into a crucial technique for *LM* detection in cerebrospinal fluid in recent years since Yao et al. originally reported three cases of acute or subacute meningitis with negative cerebrospinal fluid culture but confirmed as LMM using NGS in 2016 [25,26]. We looked through six articles published on Pubmed since 2016 using the keywords “*Listeria monocytogenes* meningitis” or “*Listeria monocytogenes* & next-generation sequencing.” Moreover, we reviewed and discussed them (Table 2) to expect these cases. A total of 19 patients were featured in the six articles, each with different clinical presentations, such as fever (19/19), headache (11/19), diarrhea, nausea, vomiting (13/19), meningeal signs (14/19), coma (or consciousness disturbance, 11/19), hyperspasmia (5/17), and others. Intracranial bleeding, limited abduction of both eyes, hiccups, poor mental health, dysarthria, diplopia, and other symptoms were experienced by individual patients. All patients had a positive result of CSF mNGS within 2–4 days. Eight of 19 patients had a negative result of CSF culture. These results are similar to our case.

**Table 2:** Reports of NGS in the diagnosis of LMM from 2016 to 2023

Reference	Age	Gender	Clinical manifestations							Duration of CSF culture and results	Duration of CSF mNGS and results	Number of *LM* sequences detected by CSF mNGS	Antimicrobial therapy	Days of targeted antibiotic therapy	Total course of the disease (days)	Outcome
			Fever	Headache	Diarrhea, nausea, vomiting	Meningeal signs	Coma or consciousness disturbance	Hyperspasmia	Others							
Li et al. 2019 [30]	1	Female	+	−	+	Unknown	−	−	Unknown	4 days, positive	2 days, positive	2,561	Meropenem + amoxicillin sulbactam	Unknown	83	Recovered
	2	Female	+	−	+	Unknown	+	+	Unknown	5 days, positive	3 days, positive	1,011	Meropenem + vancomycin	Unknown	23	Recovered
	9	Male	+	+	+	Unknown	−	−	Unknown	7 days, positive	2 days, positive	8	Meropenem + metronidazole	Unknown	28	Recovered
Li et al. 2022 [9]	32–83	Five males and 1 female	6/6	3/6	2/6	6/6	4/6	2/6	Unknown	91 h, positive	47 h, positive	404	Five patients: meropenem + penicillin G + sulfamethoxazole tablets (SMZ), and 1 patient: linezolid + etimicin + SMZ (because of penicillin allergy)	Unknown	Unknown	Five completely recovered and 1 neurological sequelae
Yu et al. 2023 [8]	23–61	Three males and 2 females	5/5	5/5	5/5	4/5	4/5	2/5	Limited abduction of both eyes, hiccups, poor mental health	Unknown, 4 negative + 1 positive	2–4 days, positive	118–1,997	Penicillins and/or meropenem	15–37 days	Unknown	Three significantly improved and 2 fewer improvements
Zhang et al. 2021 [11]	64	Male	+	Unknown	Unknown	+	Unknown	Unknown	Intracranial hemorrhage	Unknown, positive	Unknown, positive	118	Piperacillin	Unknown	Unknown	Recovered
Yao et al. 2016 [26]	40–66	Three males	3/3	1/3	2/3	2/3	1/3	Unknown	Ataxia, dysarthria, diplopia, vertigo, hypoventilation	Unknown, negative	Unknown, positive	74, 665,486	Unknown	Unknown	Unknown	Unknown
Lan et al. 2020 [31]	66	Female	+	+	+	+	+	Unknown	Unknown	Unknown, negative	3 days, positive	486	Ampicillin + trimethoprim-sulfamethoxazole	Unknown	Unknown	Recovered

The guideline panel recommends 21 days of therapy or longer [27]. As a result, it is important to continuously review changes in the patient’s condition and associated laboratory examination data, integrate microbiological testing, quickly gauge the effectiveness of anti-infection medications, and modify anti-infection treatment plans as appropriate. Five patients got targeted antibiotic therapy for 15–37 days in the literature review, presumably as a result of more serious morbidities that prolonged antibiotic therapy. tNGS is a method of sequencing which can detect a group of organisms [28]. As a result, in our case, tNGS was utilized twice following treatment for pathogen detection, effectively evaluating the treatment’s impact on patients and providing support for extended antibiotic therapy.

In the reviewed literature, the majority of patients were given meropenem and other antibiotics. After treatment, most patients (13/19) recovered, while three (who had severe disease) had fewer improvements or neurological sequelae. In our case, the antibiotic protocol resulted from carefully considering the guideline, literature, and the patient’s condition. However, some studies had shown that *LM* strains isolated from food have developed antibiotic resistance [29]. Susceptibility testing is still required for patients with *LM* infection in the future.

## 5 Conclusion

*LM* can be detected more quickly and accurately using mNGS and tNGS, which are also useful for diagnosing and monitoring LMM. They can offer compelling justification for extending antibiotic treatment.
